# Influence of Pomegranate Appearance Attributes on Consumer Choice, and Identification of Barriers and Drivers for Consumption

**DOI:** 10.3390/foods12203803

**Published:** 2023-10-17

**Authors:** Ana Pons-Gómez, Carlos Albert-Sidro, Julián Bartual, Ferrán Yuste, Cristina Besada

**Affiliations:** 1 Sensory and Consumer Science Group, Postharvest Department, Valencian Institute for Agricultural Research, CV-315, Km. 10.7, 46113 Valencia, Spain; 2 Agricultural Experiment Station of Elche, CV-855, Km. 1, 03290 Alicante, Spain; bartual_jul@gva.es

**Keywords:** skin colour, calyx shape, fruit shape, consumer, preferences, inconvenience, healthy properties, seeds

## Abstract

In a prepurchase situation, consumers base their choice decision on external fruit characteristics, from which they infer internal characteristics. This study investigates consumer preference for pomegranate appearance using a choice-based conjoint analysis with 320 participants. We created 27 images of pomegranates that differed in varietal characteristics: colour (yellow, bicoloured, and red), shape (round, oval, and flattened), and calyx shape (open, semi-open, and closed). Colour was by far the most important factor for consumers, followed by fruit shape and calyx shape. Two preference profiles were identified. Most consumers liked bicolour and red pomegranates equally, and rejected yellow ones, while a smaller group concentrated their choice on bicolour pomegranates. In terms of fruit and calyx shape, oval and flattened fruit and open calyx were the most preferred by both consumer groups. Barriers and drivers for consumption were also investigated. There is still plenty of room to increase pomegranate consumption. Greater availability of pomegranates and ready-to-eat arils in grocery shops, obtaining new unseeded/easier-to-peel varieties, and providing a sensory label would help to overcome current barriers. Marketing campaigns should focus on a pomegranate’s health benefits and its versatility in consumption.

## 1. Introduction

The offer of pomegranate fruit (*Punica granatum* L.) and derived products has grown considerably in recent decades, mainly due to growing evidence demonstrating the nutritional and beneficial properties of pomegranates, which has led them to be considered functional food. Pomegranates are rich in bioactive compounds, and it has been demonstrated that they have high antioxidant activity levels compared to other fruits. All this exerts important protective action on consumer health by alleviating oxidative stress and protecting the human body against related diseases [1,2,3].

Pomegranates are widely grown in Spain, which is the first producer in Europe, and the first exporter in the Mediterranean Basin. In 2020, Spanish cultivation covered an area of about 5327 ha, with production accounting for 79,183 tn [4], mainly located in southern regions of the country. 

The main cultivars traditionally grown in Spain are the sweet and soft-seeded ‘Valenciana’ and ‘Mollar’. These two varieties have bicolour skin with pale red tones on a yellowish background. ‘Mollar de Elche’ obtained DOP recognition from Europe in 2016 [5]. In the last few decades, new foreign varieties have been grown in Spain for both internal and export markets. This is the case of ‘Wonderful’ from the USA and ‘Acco’ from Israel. These varieties are sourer than traditional Spanish varieties, and their skin is characterised by homogenous and intense red tones. 

Consumers’ experiences of purchasing pomegranates must inevitably be affected by the extended offer with varieties originating from other countries, which not only have a different taste but also look different from traditional varieties. In fact, marketers and researchers suspect that the introduction of these new varieties may have confused some consumers because the characteristic sweetness that consumers have associated for years with the taste of pomegranates is not present in recently introduced varieties.

In the current context, acquiring more profound knowledge about consumer behaviour regarding pomegranates is essential to preserve consumers’ fondness for this fruit and to extend its market.

Answering questions such as when, why, and why don’t people consume fruit is essential for promoting healthy habits in the population as well as from a commercial point of view. In recent decades, many studies have focused on answering such questions [6,7,8,9]. Most of these studies have approached fruit and vegetables as a generic food type, but in recent years, attention has been paid to understanding consumer response to specific fruits, such as citrus fruits or nuts [10,11,12], or to different types of fruit preparations, such as dehydrated, ready-to-eat fruit, etc. [13,14]. Now is the time to acquire such knowledge about consumer behaviour regarding pomegranates.

Previous studies have demonstrated that one of the main reasons for consuming different fruits is taste [10,11]. However, it is important to bear in mind that in a prepurchase situation, prior to the experience of tasting the fruit, consumers must base their choice almost exclusively on external appearance. Thus external characteristics are a decisive factor for deciding to purchase or not and, hence, the need to guide pomegranate breeders about not only consumers’ taste preferences [15,16] but also about appearance preferences. Pomegranate breeding programmes have been established in the last few decades in pomegranate-growing countries such as China, Iran, India, Turkey, Israel, and Spain [17,18]. Knowing consumer preferences for fruit appearance is particularly relevant information for these programmes to obtain varieties with potential success in markets.

Skin colour, fruit shape, and calyx shape are the main morphological characteristics that are cultivar-dependent, and they markedly affect pomegranate appearance. After characterising morphological characteristics of 204 wild accessions from north Iran, Khadivi and Arab [19] described three skin-colour types (yellow, yellow-red, and red), three calyx shapes (closed, semi-open, and open), and two fruit shape types (round and oval). Our expertise work with different varieties from the germplasm bank and breeding programme of the Valencian Institute of Agricultural Research (IVIA)/Elche Agricultural Experimental Station (EAAE) (Spain) led us to differentiate the oval varieties into two different subgroups: fruit that is slightly flattened (slightly wider than high) and fruit with a very flattened shape (much wider than high).

In this context, the aim of this study was to identify the main barriers, drivers, and consumption contexts of pomegranates and to investigate the impact of different appearance attributes (skin colour, calyx shape, and fruit shape) on consumer choice. We also investigated to what extent consumers are able to associate the main commercial varieties with their differentiating sensory attributes.

## 2. Materials and Methods

This study was based on consumers completing an online questionnaire that was implemented using Google Forms. Participants were recruited by street surveys employing a tablet device. They received a small gift as a reward for their collaboration and were requested to invite family, friends, and/or colleagues to participate by forwarding the online survey link. A total of 320 people participated in the study. Of these, 296 were approached directly on the street and invited to complete the questionnaire in a specially adapted room (12 individual tables with chairs and daylight) on the street level of a city centre building. Presumably the remaining 24 participants completed the questionnaire at home. Participants were instructed to take as much time as necessary to answer the questionnaire. Their age distribution was as follows: 133 participants were 18–30 years old, 95 were 31–50 years old, and 92 were over 50 years old. Of the participants, 198 were women, 118 were men, and 4 self-identified as non-binary.

The questionnaire was structured into three parts (Table S1): (1) in the first part, the influence of different pomegranate appearance factors on consumer choice was investigated using a conjoint analysis; (2) in the second part, the main barriers and drivers for pomegranate consumption, and the main consumption contexts, were researched; (3) finally, to what extent participants were aware of the sensory properties of different commercially important pomegranate varieties was studied.

### 2.1. Using a Conjoint Analysis to Determine the Influence of Pomegranate Appearance Factors on Consumer Choice

High-quality colour images of pomegranates displaying different appearance characteristics were taken by a professional photographer. The evaluated appearance factors and their levels were: skin colour (yellow/bicolour/red), fruit shape (round/oval/flattened), and calyx shape (closed/semi-open/open) (Table 1). Bicolour skin corresponds to red tones on a yellowish background.

**Figure 1:**
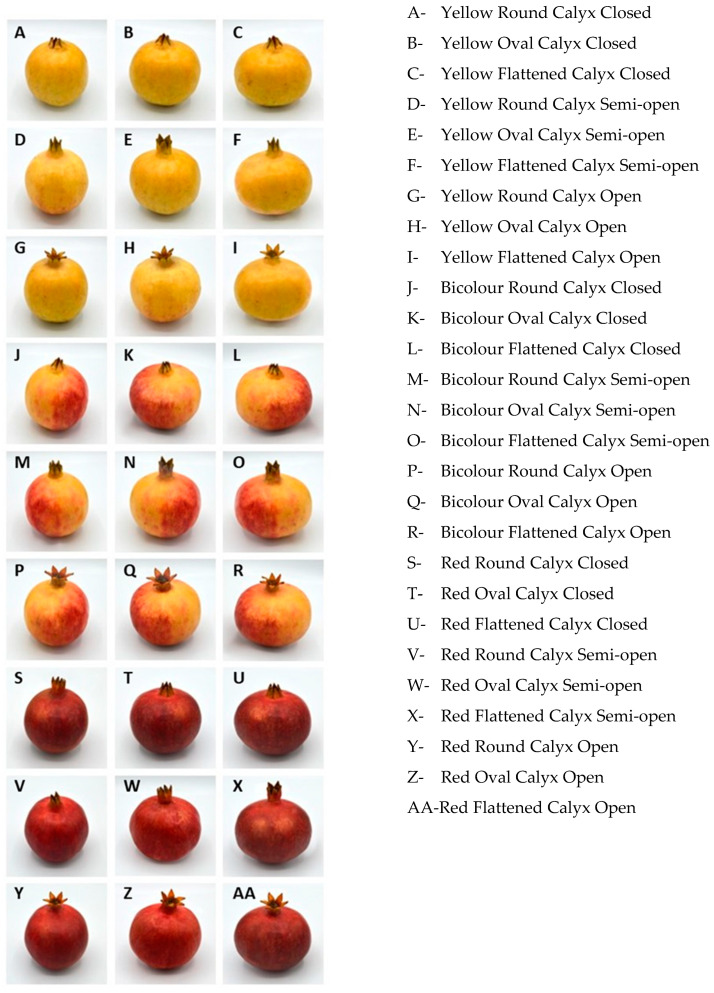
Images of pomegranates used to evaluate the effect of external appearance factors on consumer choice.

The initial images were taken of the varieties belonging to the germplasm bank and breeding programme of IVIA/EEAE. Pictures were taken on the same day that the fruit was collected. By using image editor software, 27 images were generated, which combined all the possible combinations of the three appearance factors (3 × 3 full factorial design) (Figure 1). This approach is supported by previous studies, which have demonstrated the validity of using images instead of real products [20].

The choice-based conjoint (CBC) analysis relies on the selection of the preferred product among several alternatives presented at the same time. This task is similar to making a choice among different products that consumers perform in their everyday lives while shopping in real markets [21]. The CBC analysis was, therefore, selected to evaluate the influence of different pomegranate appearance factors, and their combination, on consumer choice.

Participants were presented with a 9-choice set consisting of 3 photographs of pomegranates. Triads were displayed successively on a tablet screen. Product presentation was randomised across participants at both the choice-set level and the product level within choice sets. The XLSTAT 2022.2.1 statistical software (Addinsoft, New York, NY, USA) was used to generate the CBC design.

At the beginning of the task, consumers were asked to indicate: “Imagine you go to your usual greengrocer to buy pomegranates either for yourself or because someone else has ordered them. We are going to show you different types 3 by 3, and we want you to tell us in each case which one you would choose if all three types were available in your greengrocer shop”. The participants responded by clicking on one of the three alternatives.

### 2.2. Barriers, Drivers, and Contexts for Pomegranate Consumption

In the second survey section, consumers were asked if they consumed pomegranates on a regular basis by answering Yes/No. Those consumers who stated they did not consume pomegranates were asked about their reasons for not doing so. To this end, consumers were given a list of reasons and were asked to select all those they considered applicable in each case. The multiple-choice list included the following options: ‘They do no usually look fresh, bad quality (old, damaged) and I think they will not be good’, ‘Very expensive’, ‘I don’t know how to eat them’, ‘I haven’t tasted them and they do not appeal to me’, ‘I don’t recognise the ones I like’, ‘I don´t like their taste’, ‘I don’t eat them whole and what remains spoils’, ‘They are not available where I usually shop’, ‘Seeds bother me’, ‘My family is not in the habit of eating them’, ‘They are difficult to peel and prepare’, and ‘Messy when eating’.

Those consumers who stated being habitual pomegranate consumers were asked about the reasons why by answering the following multiple-choice question: ‘What are the main reasons why you usually consume pomegranates? Check all that apply’. The available options were: ‘They are cheap fruit’, ‘They remind me of Christmas’, ‘It amuses me to peel them’, ‘I find arils colour very appealing’, ‘They are local fruit’, ‘They are especially good for health’, ‘I really like their taste’, ‘If I don’t eat them whole, what’s left holds up well’, ‘Keep well for many days’, ‘They can be prepared in many ways’, and ‘As a tradition, my family has always eaten them’.

It has been reported that the final decision to buy or consume a particular food depends as much on the expected context of use as on the intrinsic properties of the product [22]. Thus after indicating the reasons for consuming, participants were asked about the consumption contexts by means of the following multiple-choice question: ‘When and how do you usually eat them? Check all that apply’. The options for responding were: ‘I buy grains’, ‘At breakfast’, ‘As part of school lunch boxes for children’ ‘To make desserts/cakes’, ‘As a snack between meals’, ‘I eat them with yogurt’, ‘In salads and cold dishes’, ‘As a dessert at lunch or dinner’, ‘I only eat them if they are given to me’, ‘I buy juice’, ‘I make juice/smoothies with them’, ‘I peel them, store grains in a container and eat a few every day’, and ‘I buy whole fruit’.

As the specific literature on this matter for pomegranates is lacking, all three lists of options (barriers, drivers, and contexts) were designed based on the previously existing literature for other specific fruits or for fruit as a generic group [23,24,25,26,27], as well as on previous author experiences. Then a group of eight consumers participated in a session to generate the final list of terms to be included in the questionnaire. Four of them were habitual consumers and the other four were not consumers. Each subgroup consisted of two women and two men of different ages. They were asked to write down all those drivers/barriers to consumption and the contexts in which they would consume pomegranates. They could use the contexts/situations on the preliminary list but were urged to suggest new options that were relevant to finalise the lists. Thus for example, ‘they remind me of Christmas’ was a driver added to the list in this phase of the study, as was the context ‘I only eat them if they are given to me’. None of these eight consumers participated in the online questionnaire.

On the three lists (barriers, drivers, and consumption contexts), the order of presentation of the different options was randomised across participants, and the ‘Others’ option was included at the end so that participants could add any other missing reason.

### 2.3. Consumer Awareness of the Sensory Properties of Different Varieties

The final task of the questionnaire attempted to simulate a prepurchase situation in which consumers have to decide which variety to buy without tasting it. We wished to investigate the extent to which consumers are aware of the sensory characteristics of the main pomegranate varieties and can apply this awareness to guide their choices. For this purpose, participants were simultaneously presented with pictures of the ‘Mollar’, ‘Valenciana’, and ‘Wonderful’ varieties, including the variety name. The ‘Origen Spain’ claim was simultaneously presented (Supplementary Figure S1). They were given the following instructions: “Please indicate which of the varieties you see in the pictures you consider to be sour/sweet pomegranate varieties”. Half of them were asked to indicate ‘the sour varieties’, and the other half were asked about ‘the sweet varieties’. The order of presentation of the three pictures was randomised across participants.

Afterwards, participants answered some demographic questions, such as their gender and age.

### 2.4. Statistical Analysis

The fundamental premise for the CBC analysis is that a product can be understood as a set of attributes from which utility (or value) is gained. The CBC design allows one to deduce the relative values of the different attributes to the overall purchase decision. The multinomial logit regression analysis is used to do this, and in such a way that a separate value, named ‘utility’, is calculated for each attribute of a product. Positive utility values indicate that the attribute contributes positively to the likelihood of purchase, while negative utility values indicate a negative contribution [21]. For example, the utility of a pomegranate with red skin, a round shape, and a closed calyx can be estimated by summing the utilities pertaining to red skin, to a round shape, and to a closed calyx. Once the utility associated with each attribute has been obtained, the relative importance attached to each attribute can be calculated.

Analysis of choice data was made by multinomial logit model regression using the XLSTAT 2022.2.1 statistical software (Addinsoft, New York, NY, USA). The model included the main effects (i.e., individual attributes) and the interaction between them. This provided a more in-depth understanding of how the experimental factors determined consumer responses. The utility value for each level or interaction was obtained.

Additionally, individual utilities were obtained for each consumer by logit model regression considering only the main effects. A hierarchical cluster analysis (HCA) was applied to the individual utilities to identify groups of consumers who had similar choice patterns. Euclidean distances and Ward’s aggregation method were considered.

## 3. Results and Discussion

### 3.1. Influence of Appearance Factors on Consumer Choice

Our results revealed that skin colour was the most determinant factor in pomegranate choice. Thus, at the time of making their choice decision, participants attached 68% importance to fruit colour, 25% to shape, and only 7% to calyx shape. The relevance of colour on consumer choice of the different fruit types has been previously described: dark skin cherries were preferred by American consumers [28], while green areas on mandarin skin were penalised by Spanish consumers [29]. The importance of colour for consumer choice has also been described for pears but, in this case, it was the second most important factor behind fruit shape [30].

**Figure 2:**
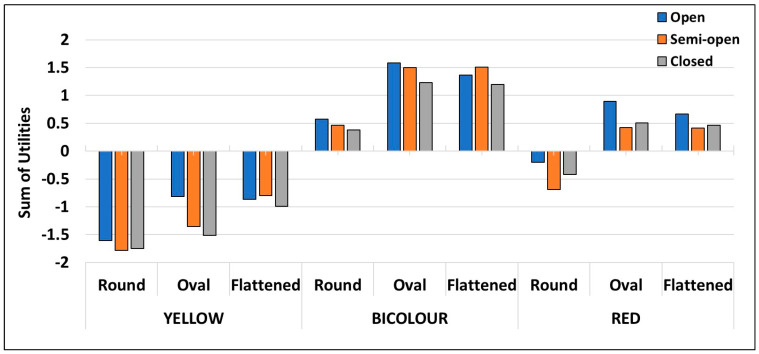
Global effect (sum of utilities of the individual factors and their interactions) of external appearance factors on pomegranate choice. The total number of participants in the conjoint test was 320.

The CBC analysis allows not only the effect of the main factors but also the interactions between such effects to be investigated. Taking into account the whole dataset, the utilities for the different combinations of factors were calculated (Figure 2). It reflects the marked importance that participants attached to fruit colour. Thus the attributes combination, including the yellow colour, had negative utilities in all cases. On the contrary, bicolour skin was always linked with positive utilities. Halfway between both, the red colouration utility depended on fruit shape; thus oval and flattened pomegranates with red skin showed positive utilities, while red colouration combined with a round shape had a negative impact on choice. The impact of shape was also evident for bicolour fruit, with lower utility values for round than for flattened and oval fruit.

According to their lesser relative importance, the effect of calyx shape on utility values was less evident. However, if we focus on red fruit, irrespective of fruit shape, the fruit with an open calyx was preferred over semi-open and closed ones.

Previous studies have reported that calculating the utilities for each factor level for every participant and applying an HCA to such a dataset allows one to gain a better understanding of consumer behaviour by identifying different choice profiles [29]. This approach was followed in the present study, and it revealed two different choice profiles among participants: cluster 1, which included the vast majority of participants (70% of them); cluster 2, which included the remaining 30% (Figure S2).

**Figure 3:**
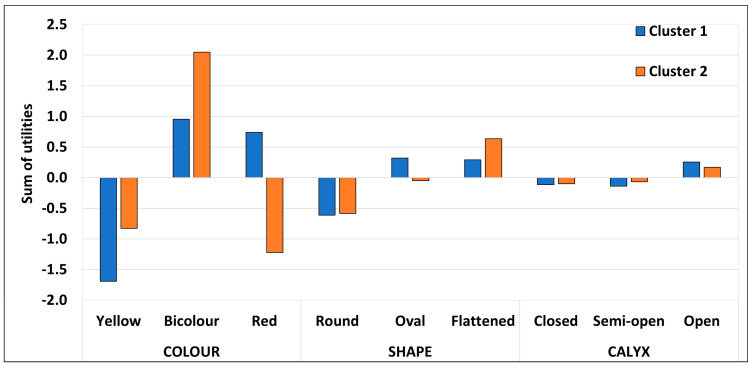
Global effect (sum of utilities of the individual factors and their interactions) of external appearance factors on pomegranate choice for the two identified clusters. The total number of participants in the conjoint test was 320.Cluster 1 included 70% of them and Cluster 2 the remaining 30%.

The values of the utilities for the different factor levels for each cluster are depicted in Figure 3. The main difference among clusters was due to participants’ colour preferences. On the one hand, the consumers in cluster 1 clearly rejected yellow pomegranates, while the red and bicolour fruit were equally well liked. On the other hand, the consumers in cluster 2 rejected both yellow and red pomegranates and showed a marked preference for bicolour ones.

**Figure 4:**
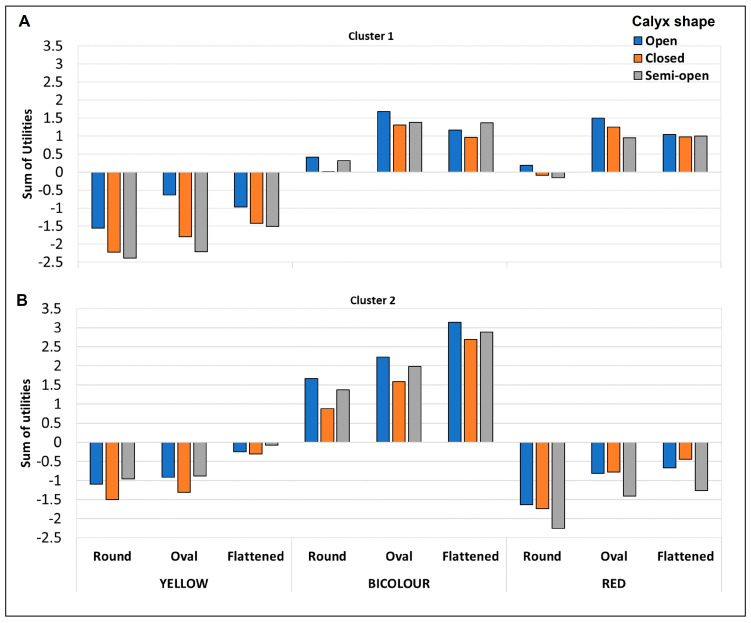
Global effect (sum of utilities of the individual factors and their interactions) of external appearance factors and all their combinations on pomegranate choice for the two identified clusters ((**A**) Cluster 1, (**B**) Cluster 2). The total number of participants in the conjoint test was 320. Cluster 1 included 70% of them and Cluster 2 the remaining 30%.

After establishing the main differences among clusters, for a more detailed study of the behaviour of each group of participants, the utilities of all the combinations of factors were calculated. They are shown in Figure 4A,B. The significance of each factor and their interactions appear in Table 2. Colour and fruit shape were significant factors for both consumer profiles, while calyx was significant only for the choice profile from cluster 1. Regarding the interaction among factors, which indicates the extent to which participants’ preferences for one factor level were conditioned by the level of the other factors, colour ∗ calyx was significant for both clusters of participants, while colour ∗ shape was significant only for cluster 1. The interaction among the three evaluated factors was significant in both cases.

As previously mentioned, the participants grouped in cluster 1 showed clear preferences for bicolour and red fruit and rejected yellow ones (Figure 4A). Fruit shape was also a relevant factor, mainly for those fruit for which they liked colouration. For the bicolour and red fruit, the round shape obtained much lower utility values than the oval and flattened ones. This effect was also observed, but was not so evident, for the rejected yellow pomegranates.

Open calyx was the preferred calyx shape by this group of consumers, followed by semi-open and closed. The lesser preferences for the closed and semi-open calyxes, compared to the open one, were observed mainly for the yellow and red fruit, of which participants preferred the fruit with an open calyx irrespectively of fruit shape.

Based on these results, we can conclude that the participants from cluster 1 liked the bicolour and red pomegranates with an oval and flattened shape and rejected the yellow and round ones. Of them, the most preferred pomegranate aspect can be described as bicolour, oval-shaped, and having an open calyx.

The cluster 2 participants showed clear preferences for bicolour pomegranates and rejected both the yellow and red ones (Figure 4B). Of the bicolour fruit, the most liked were those with a flattened shape, followed by oval ones. Calyx shape was not a definite characteristic, but the higher utility values were associated with an open calyx.

It is worth mentioning that the pomegranate characteristics preferred by the consumers in cluster 2 were mainly those of the traditional Spanish ‘Mollar’, and ‘Valenciana’ varieties. A study performed by Gamble et al. [30], in which preferences of New Zealander and Australian consumers for pear appearance were evaluated, concluded that consumers looked for pear attributes that were familiar to them. Thus it is possible that the preference pattern of the participants in cluster 2 was linked with being more familiar with the attributes of traditional Spanish varieties compared to the attributes of the recently introduced varieties, such as the red colour of ‘Wonderful’ or ‘Acco’.

### 3.2. Barriers, Drivers, and Consumption Contexts

The questionnaire designed for this study guided participants to different sections depending on their response to the consumption question. Thus we investigated the (1) consumption barriers of those participants who stated that they did not consume pomegranates and (2) reasons for consuming and the consumption contexts of pomegranate consumers.

The first result to comment on is that the percentage of participants who stated that they consumed pomegranate (45%) was lower than those who did not (55%). Such data leave much room to increase pomegranate consumption and highlight the importance of this study. A better understanding of the barriers and drivers for pomegranate fruit consumption will facilitate the more effective targeting of commercial strategies to make this fruit more appealing to consumers.

#### 3.2.1. Consumption Barriers

**Figure 5:**
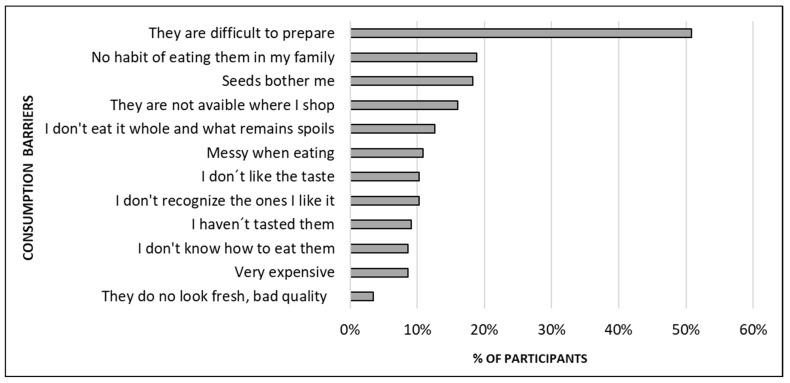
Main barriers to consuming pomegranate for the 176 respondents who reported not being habitual consumers. Participants were instructed to select all the options that applied to them.

The main barriers to pomegranate consumption are shown in Figure 5. The results are quite revealing because ‘they are difficult to prepare’ stands out among other reasons for not consuming. Of less importance, but still worthy of mention for being indicated by more than 10% of the participants who did not consume pomegranates, were identified barriers such as ‘my family is not in the habit of eating them’, ‘seeds bother me’, ‘they are not available where I usually shop’, ‘I don´t eat them whole and what remains spoils’, ‘messy when eating’, ‘I don’t like their taste’, and ‘I don’t recognise the ones I like’.

Most of the existing studies that have investigated barriers to fruit consumption have approached fruit and vegetables together as a single food category [31], while only a few have evaluated fruit separately [23]. In both cases, lack of convenience was identified as a major barrier to fruit consumption. It has been linked to a lack of time to prepare fruit and consumers finding it more convenient to eat processed food. However, not all fruits take the same amount of time to prepare, and this can influence consumer choices. For example, Tarancón et al. [13] reported that ‘easy-to-peel’ fruit, such as mandarins or bananas, were preferred by consumers in most daily contexts, while cutlery-needed fruit, which takes longer to prepare, was less often chosen. Undoubtedly, the preparation of pomegranate (peeling and separating arils) requires cutlery and takes longer than preparing most other fruit. All this explains why our results showed the inconvenience of preparation as the main barrier to consumption. Another aspect related to inconvenience is the unpleasantness associated with the staining of hands/clothes during preparation, which has also been reported as a barrier for very juicy fruits, such as oranges [11].

The second most important reason for not consuming pomegranates was ‘my family is not in the habit of eating them’. It should be noted that pomegranate production in Spain and other Mediterranean countries has significantly increased in recent years, but neither its cultivation nor its consumption has been so widespread in previous decades. This may be the reason why there are still households where this fruit does not form part of the shopping basket because it has been shown that preference and consumption of fruit and vegetables in adulthood are closely linked with their consumption in childhood [32]. Lack of availability was also identified as a barrier to establishing a regular consumption habit.

Regarding fruit organoleptic properties, the most relevant barrier was the inconvenience caused by seeds, while ‘I don’t like their taste’ was less frequently mentioned. The presence of pips, seeds, or stones seems to be a barrier to consuming several fruits, such as cherries or grapefruit [23]. As regards pomegranates, sensory studies that have evaluated consumer preferences report that seed hardness is the main characteristic responsible for seeds being inconvenient for consumers [33], and consumers show preferences for soft-seeded varieties. Another aspect that has been evaluated in certain studies is the woody index, which indicates the seed weight/aril weight ratio [34].

Another barrier of certain relevance was ‘I don´t recognise the ones I like’. As mentioned in the Introduction, consumer confusion must be linked with the relatively recent introduction of new varieties with different organoleptic properties to traditional varieties. Currently, two main types of varieties are commercialised in Spain: sweet and sour ones. Some varieties have also been classified as semi-sour [35]. Unlike other fruits, like apples, for which consumers can find different varieties at the same point of sale and can choose the fruit that matches their preferences, only one type of pomegranate is usually offered in grocery stores and supermarkets. Sellers there do not pay much attention to informing consumers about the variety name unless it is certified by the ‘Mollar de Elche’ DOP [5].

A recent study performed in Spain revealed consumers’ interest in a label that provided information about sensory fruit properties [36]. Despite this study not asking consumers specifically about the type of information that they would like to know about pomegranate, the sweetness and sourness level was reported as demanded information for most evaluated fruit. It is very likely that a sensory label would help consumers to identify the pomegranate varieties that they like. In addition to sweetness and sourness, seed hardness seems to also be relevant information to be included on labels.

Taking into account all the barriers herein identified, to overcome them, different, but complementary, strategies are likely to be needed. The first one would be marketers to ensure greater availability of pomegranates at groceries and supermarkets as both fresh fruit and ready-to-eat arils. Availability of ready-to-eat arils may enhance pomegranate consumption among peeling- and dressing-shy consumers and may be a good option for general consumers whose time is limited. Much progress has been made in the last two decades in obtaining ready-to-eat arils [37,38,39]. Despite this product starting to appear on the shelves of some supermarkets, according to a recent review by Bhatia and Asrey [40], prolonging the shelf life of this product is still a challenge. Research is still underway to control the main shelf-life limiting factors, mainly browning and loss of organoleptic quality.

Adding a sensory label that helps consumers identify those varieties with the sensory properties that they would like would probably have a very positive impact on pomegranate consumption. The sensory label should be included for both fresh pomegranates and ready-to-eat arils and should include information about sweet and sour levels, which is the major difference among varieties [35], and also about seeds.

Seed properties should also be a main aspect for breeders, and efforts should be made to obtain unseeded varieties or varieties with very soft and small seeds that are as imperceptible as possible.

#### 3.2.2. Drivers and Contexts for Consumption

**Figure 6:**
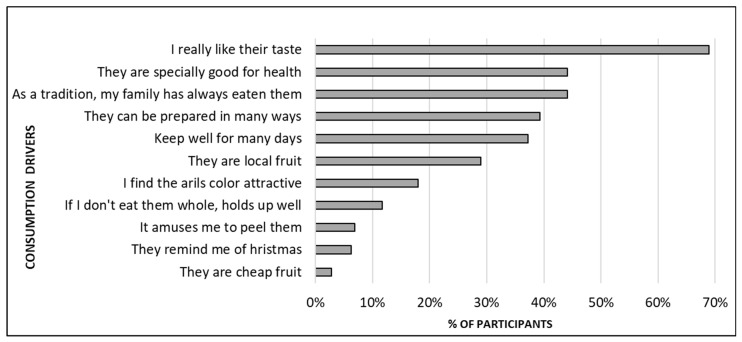
Main drivers of pomegranate consumption of the 144 participants who reported being habitual consumers. Participants were instructed to select all the options that applied to them.

The core drivers for pomegranate consumption were identified as taste, followed by health properties and family habits (Figure 6). The considerable importance that participants attached to taste and health benefits was in accordance with previous studies, in which both factors were identified as the main drivers for the consumption of a specific fruit, mainly citrus fruit [10,11]. Regarding the importance of family habits for eating a specific fruit, different studies have reported that food choice in adults is highly influenced by what they were taught at home in the past, and people continue eating according to their parents’ habits when they leave their family home [41,42,43].

Consumer interest in local food has steadily increased in the past few years and, accordingly, pomegranate as local food appears among the main identified consumption drivers. The participants of this study were questioned in Valencia, a city allocated not too far (≈150 km) from the main pomegranate producer area of Spain. Thus, pomegranate was considered a local food. According to the review by Feldmann and Hamm [44], perceptions of local food being of better quality and taste than non-local food were the main reasons for consumers to choose it. More trust, linked with the perception that local food is safer and easier to trace back, also has a positive influence. In addition, more altruistic attitudes, such as supporting the local economy, environmental friendliness, and farm workers obtaining better benefits, may also motivate interest in local food.

**Figure 7:**
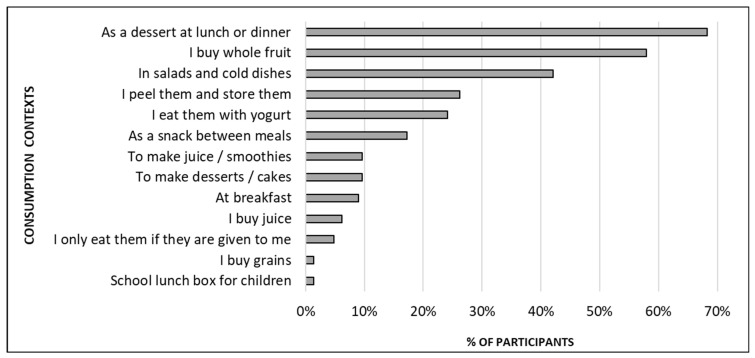
Main contexts of pomegranate consumption of the 144 participants who reported being habitual consumers. Participants were instructed to select all the options that applied to them.

Other aspects of pomegranate that motivate consumers to buy it are associated with its versatility in terms of use and its durability both before and after the fruit is cut. Such versatility was corroborated after evaluating the consumption contexts (Figure 7). Thus consumers started using it as a dessert for main meals, to prepare salads, to eat with yoghurt, or to eat it as a snack between meals. Most consumers mentioned buying whole fruit, and some indicated that they prepare it and then store arils. Although the inconvenience of preparing fruit was identified as the main barrier to consumption (Figure 6), only a very low percentage of participants stated that they buy ready-to-eat arils. A number of factors must be involved in such a decision, and the lack of availability of ready-to-eat arils in green groceries and supermarkets is likely to be one of them. It is also important to bear in mind that there are still barriers to consuming ready-to-eat fruit. Two important barriers identified by Tarancón et al. [13] were high prices and the use of plastic packaging. In any case, when evaluating together the results of the barriers and consumption contexts obtained in the present study, one doubt arises: why do people not buy ready-to-eat arils if they would overcome the inconvenience barrier? Is it only a matter of availability? Answering this question would be an interesting topic for future studies.

#### 3.2.3. Awareness of the Sensory Properties of Different Varieties

In other fruits, like mandarins and apples, it has been reported that two consumer types exist: those who like sour varieties and those who like sweet ones [26,45]. In a recent sensory study, our research group observed that this pattern is also applicable to pomegranate consumers because, after almost 100 consumers evaluated twelve varieties, we identified two profiles of consumer preferences: those who like sour pomegranate varieties and those who prefer sweet ones. These results will soon be published.

Bearing these results in mind, in the third section of the questionnaire, participants were shown pictures of three of the most important commercial varieties, including one sour variety (‘Wonderful’) and two sweet varieties (‘Mollar’ and ‘Valenciana’). Participants were asked to identify those with specific sensory properties: ‘the sour ones’ or ‘the sweet ones’.

The results of this task showed that only 14% of the participants gave the correct answer, i.e., identified ‘Mollar de Elche’ and ‘Valenciana’ as sweet or ‘Wonderful’ as sour, without making any mistake; 32% correctly identified one of the sweet varieties without making mistakes. Initially, we could state that these two groups of participants know enough varieties to buy those with their preferred sensory properties, whatever they may be. On the contrary, most of the participants (54%) are at risk of selecting varieties they may not like.

These results must be discussed in two main aspects. On the one hand, it is interesting to evaluate if there is a relation between awareness of properties and consumption, i.e., if habitual consumers know varieties better than non-consumers. To do so, we evaluated if those participants who failed were the same as those who stated not habitually consuming pomegranate. The data on this showed that 76 of the 176 participants who failed, i.e., 43% of them, were habitual consumers. It seems, therefore, that being a habitual consumer does not guarantee recognising varieties for their sensory properties. Gender and age were also found to have no influence on awareness of the properties of different varieties.

The second aspect to discuss is to what extent is there a connection between the unawareness of the properties of different varieties and consumption barriers. The results of the present section explain why ‘I don´t recognise the ones I like’ was identified as a barrier to consumption. The data also revealed that there were more people who did not know how to identify varieties than those who indicated a lack of awareness as a barrier. These data suggest that a substantial percentage of people are not aware of their limitations when choosing pomegranates that match their preferences. This fact may, to some extent, be associated with the barrier ‘I don´t like their taste’, reported by 9% of the participants. Thus if consumers do not know how to select pomegranates according to the properties they like, and make a wrong choice, they may conclude that they do not like the taste of pomegranates.

## 4. Conclusions

Of the main appearance factors that depend on variety, skin colour is the most important in consumer choice decisions. This study reveals two preference profiles regarding pomegranate appearance. Seventy per cent of consumers equally liked bicolour and red pomegranates, while the rest rejected both yellow and red pomegranates and concentrated their choices on bicolour pomegranates. In terms of fruit and calyx shape, oval and flattened fruit and open calyx were the most preferred by both groups of consumers.

Only 45% of the participants were habitual pomegranate consumers. This suggests that there is still a lot of room for increasing pomegranate consumption. Moreover, the information provided herein can help breeders to set targets and the industry to take action to promote its consumption. Increasing pomegranate availability at grocery stores and supermarkets as both fresh fruit and ready-to-eat arils should be the first action to overcome consumption barriers. In addition, the availability of unseeded or less perceptible seeded pomegranates should be considered a key factor for breeding to improve consumer experiences when eating fruit. In the same vein, any improvement in the difficulty of preparation, such as obtaining varieties that are easier to peel and with arils that are easier to separate, would be welcomed by consumers. More than half the habitual consumers were unaware of the organoleptic properties of the main commercial varieties. Therefore, the addition of a sensory label with information on sweetness, sourness, and seed hardness would help to ensure that consumers choose the varieties that match their preferences. Marketing campaigns should focus on the health benefits of pomegranates and their versatility because these are key drivers for consumption.

## Figures and Tables

**Table 1:** Levels of the pomegranate’s external characteristics evaluated in the conjoint design.

Appearance Factors	Level
Skin colour	Yellow
	Bicolour (yellow-red)
	Red
Fruit shape	Round
	Oval Flattened
Calyx shape	Closed Semi-open
	Open

**Table 2:** Level of significance (*p*-value) of the different appearance factors and their interactions in pomegranate choice.

Attributes	Cluster 1	Cluster 2
Colour	<0.0001	<0.0001
Shape	<0.0001	<0.0001
Calyx	<0.0001	0.198
Colour ∗ Shape	0.033	0.100
Colour ∗ Calyx	0.046	0.031
Shape ∗ Calyx	0.527	0.880
Colour ∗ Shape ∗ Calyx	0.003	0.016

## Data Availability

Data presented in this study are available from the corresponding author upon request.

## Supplementary Materials

The following supporting information can be downloaded at: https://www.mdpi.com/article/10.3390/foods12203803/s1, Figure S1: Images of pomegranates used to evaluate consumer awareness of the sensory properties of the main commercial varieties; Figure S2: Clusters of participants based on their choices of pomegranates. Table S1: Questionnaire used in the study.
