# Supplementary material for: Influence of Pomegranate Appearance Attributes on Consumer Choice, and Identification of Barriers and Drivers for Consumption
Source: Foods. 2023 Oct 17;12(20):3803. doi: 10.3390/foods12203803 (PMC10606882; doi:10.3390/foods12203803)
Supplement: Supplementary file 1 [file foods-12-03803-s001.zip › foods-2628056-supplementary.pdf]

# SUPPLEMENTARY MATERIAL

## Influence of pomegranate appearance attributes on consumer choice, and identification of barriers and drivers for consumption

Pons-Gómez A., Albert-Sidro C., Bartual J., Yuste F., Besada C.

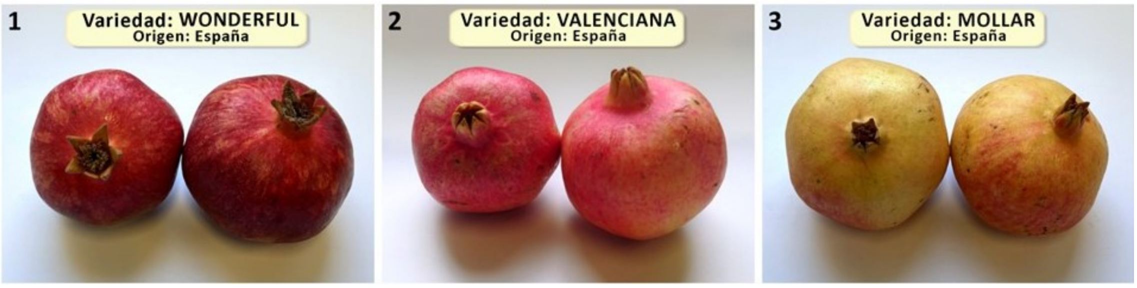


**Figure S1.** Images of pomegranates used to evaluate consumer awareness of the sensory properties of the main commercial varieties.

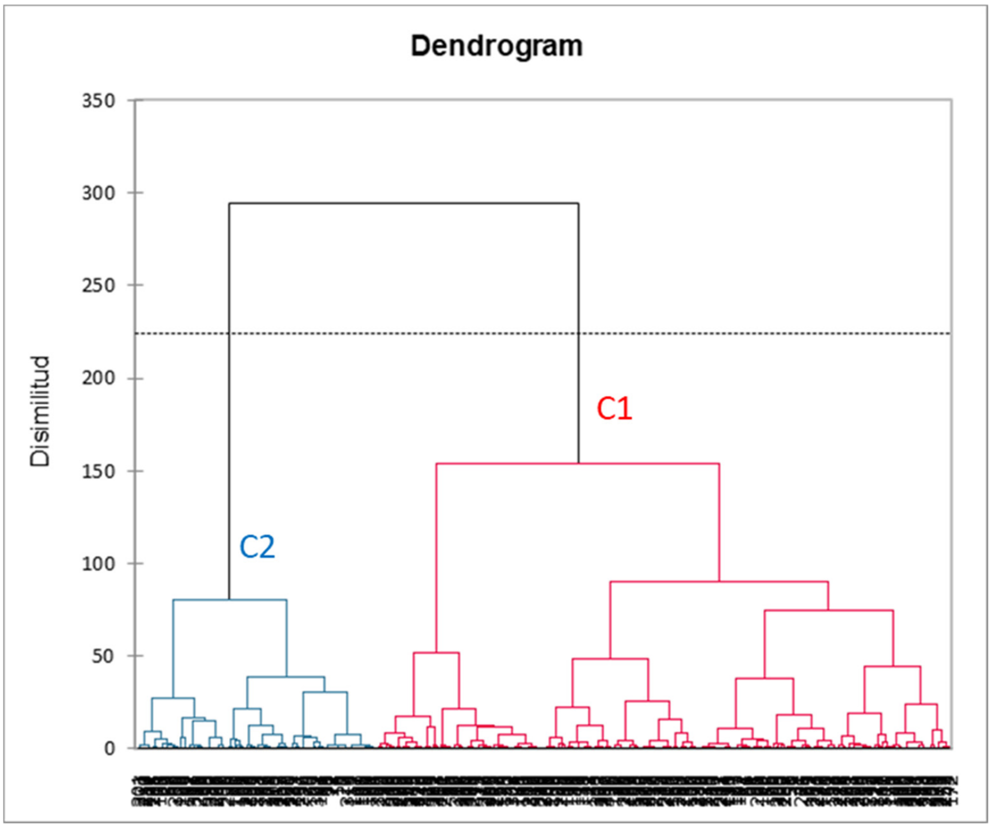


**Figure S2.** Clusters of participants based on their choices of pomegranates.

**Table S1.** Questionnaire used in the study.

We would like to invite you to participate in a study being carried out by the Valencian Institute of Agricultural Research (Instituto Valenciano de Investigaciones Agrarias). The aim of the study is to investigate consumers' preferences regarding the appearance of pomegranates.
If you agree to participate in this study, you will have to answer a questionnaire that will take about 4 minutes to complete.
Your answers will be anonymous, the only personal information you will be asked for is your gender and age range. The analysis of the results will be statistical, so no individual results will be disseminated. The results will be presented at conferences and may be published in scientific journals.
Your participation in this project is voluntary and you may decline to participate immediately or at any time during the completion of the questionnaire. By agreeing to participate, you will also be contributing to the advancement of science.
If you agree to participate voluntarily, click "Next" to begin the questionnaire.

Imagine you go to your usual greengrocer to buy pomegranates either for yourself or because someone else has ordered them. We are going to show you different types 3 by 3, and we want you to tell us which one you would choose if all three types were available in your greengrocer shop.

Which pomegranate would you choose?

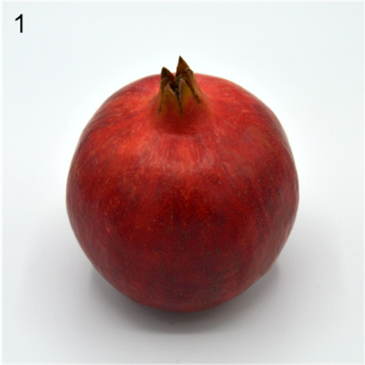

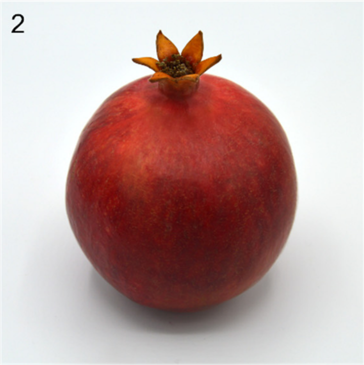

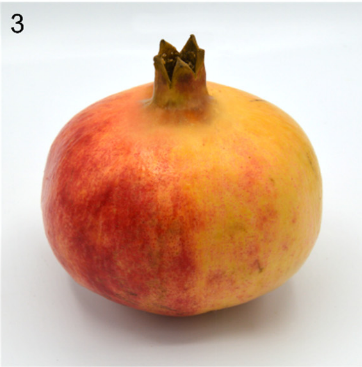


Which pomegranate would you choose?

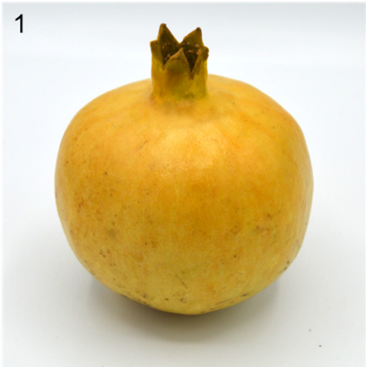

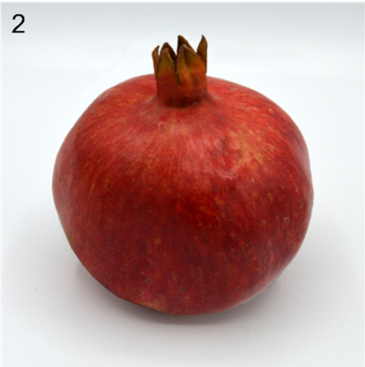

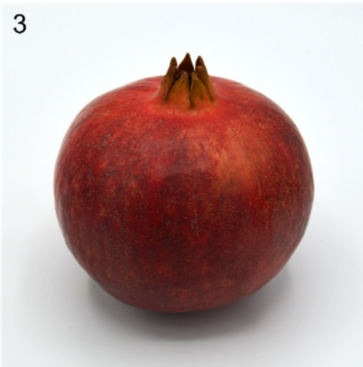


Which pomegranate would you choose?

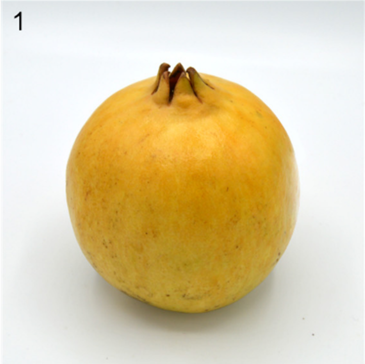

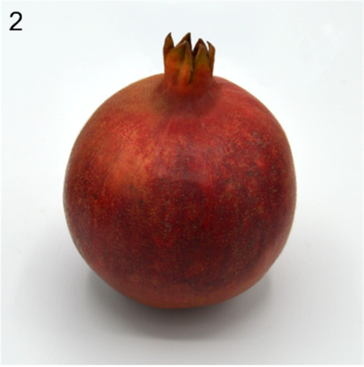

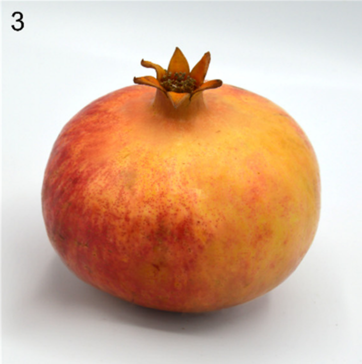


Which pomegranate would you choose?

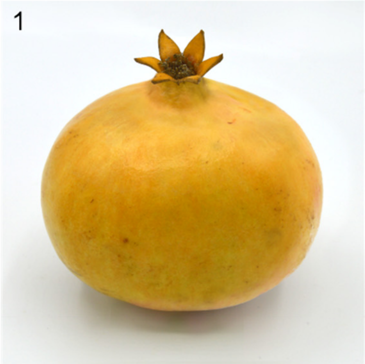

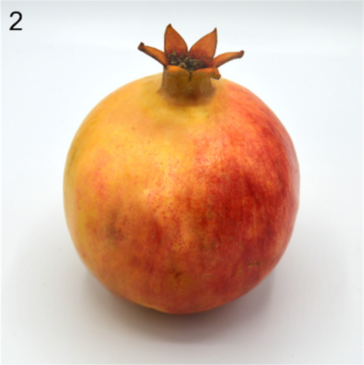

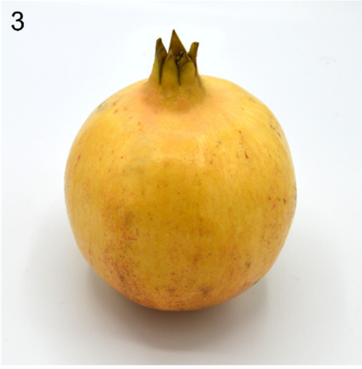


Which pomegranate would you choose?

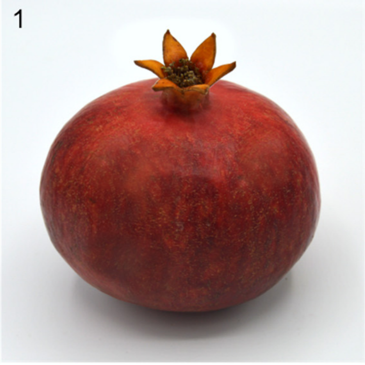

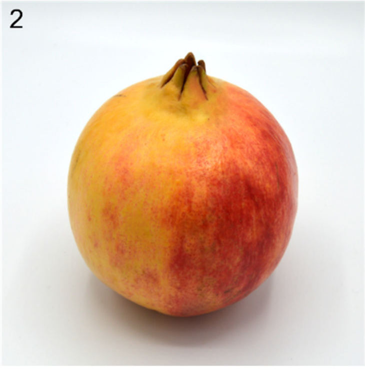

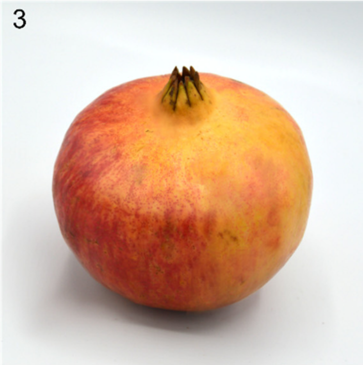


Which pomegranate would you choose?

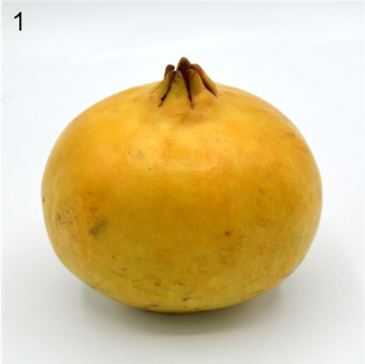

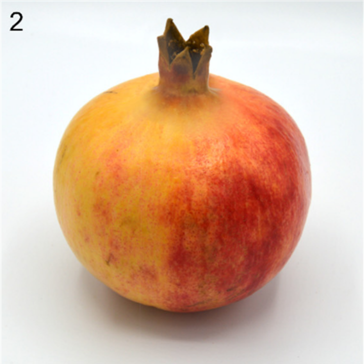

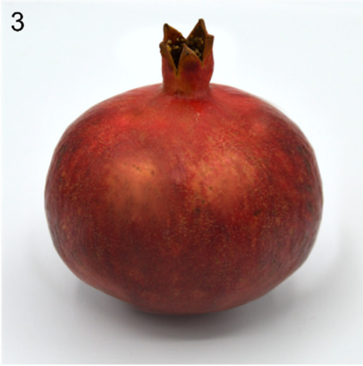


Which pomegranate would you choose?

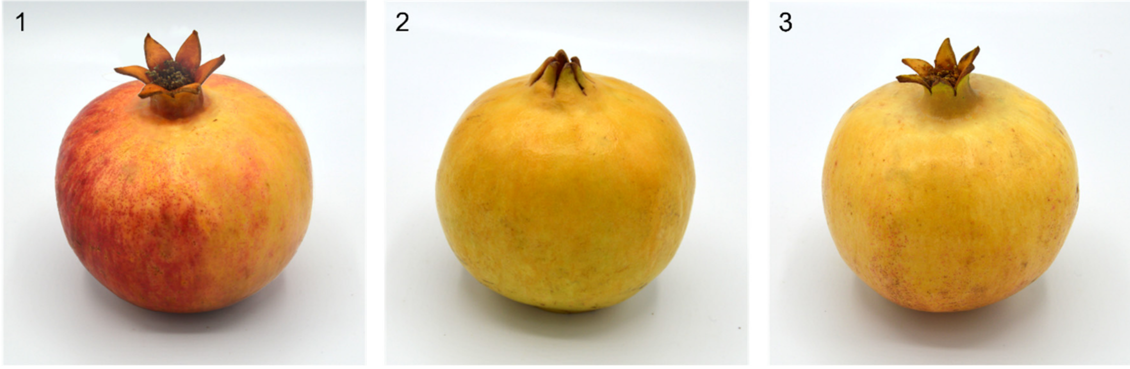


Which pomegranate would you choose?

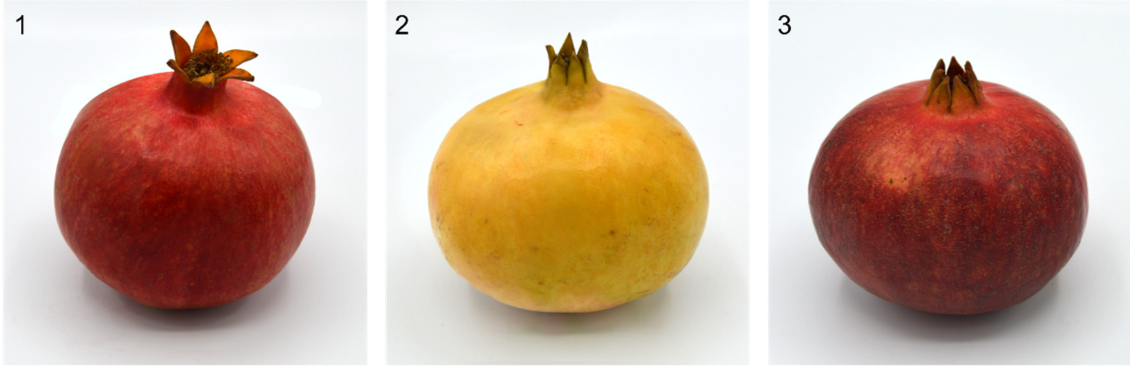


Which pomegranate would you choose?

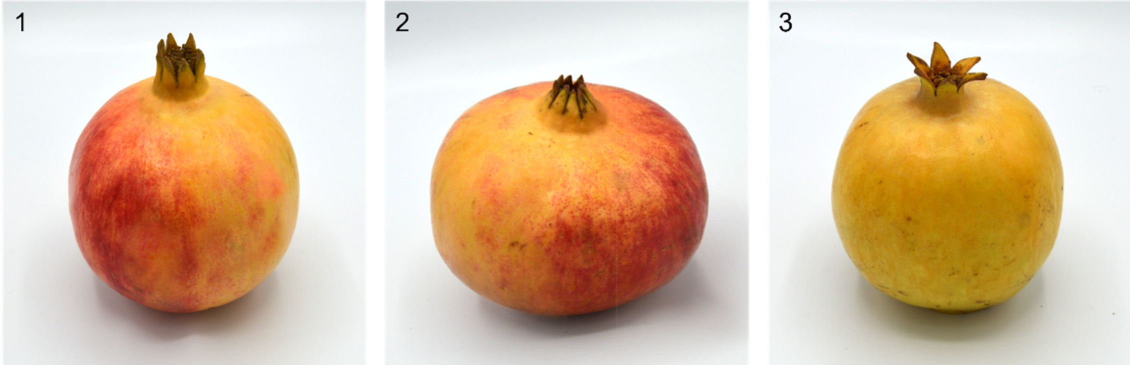


A few more questions about your pomegranate consumption

During the pomegranate season, do you regularly eat pomegranates ?

☐ Yes ☐ No

Which of these reasons do you usually not eat pomegranates? Check all that apply.

☐ They do no usually look fresh, bad quality (old, damaged) and I think they will not be good.
☐ Very expensive.
☐ I don't know how to eat them.
☐ I haven´t tasted them and they do not appeal to me.
☐ I don't recognise the ones I like.
☐ I don´t like their taste.
☐ I don't eat them whole and what remains spoils.
☐ They are not available where I usually shop.
☐ Seeds bother me.
☐ My family is not in the habit of eating them.
☐ They are difficult to peel and prepare.
☐ Messy when eating.

What are the main reasons why you usually consume pomegranates? Check all that apply.

☐ They are cheap fruit.

☐ They remind me of Christmas.
☐ It amuses me to peel them.
☐ I find arils colour very appealing.
☐ They are local fruit.
☐ They are especially good for health.
☐ I really like their taste.
☐ If I don’t eat them whole. What’s left holds up well.
☐ Keep well for many days.
☐ They can be prepared in many ways.
☐ As a tradition. my family has always eaten them.

When and how do you usually eat them? Check all that apply.

☐ I buy grains.
☐ At breakfast.
☐ As part of school lunch boxes for children
☐ To make desserts/cakes.
☐ As a snack between meals.
☐ I eat them with yogurt.
☐ In salads and cold dishes.
☐ As a dessert at lunch or dinner.
☐ I only eat them if they are given to me.
☐ I buy juice.
☐ I make juice/smoothies with them.
☐ I peel them, store grains in a container and eat a few every day.
☐ I buy whole fruit.

Please indicate all the varieties that you consider to be sour/sweet pomegranate varieties.

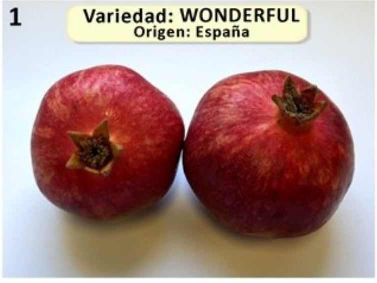

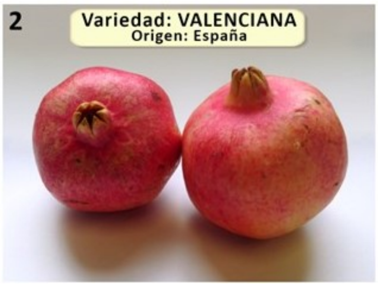

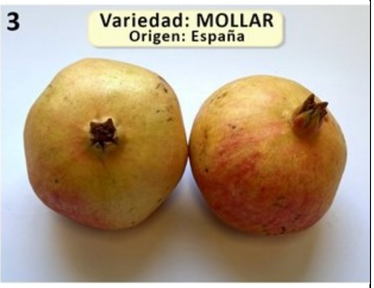


What is your gender?

☐ Female
☐ Male
☐ Non-Binary

What is your age?

☐ 18-30 years old
☐ 31-50 years old
☐ Over 50 years old

You have finished the questionnaire.
Thank you very much for your participation
